# Biochemical study on microRNAs (miR-410, miR-133 and miR-582) in Egyptian type 1 diabetic patients

**DOI:** 10.1186/s12902-025-02111-y

**Published:** 2025-12-08

**Authors:** Soha Mohamed Hamdy, Louai Mohamed Mostafa, Sherin Khamis Hussein, Shymaa E. Ayoub

**Affiliations:** 1 Department of Chemistry, Faculty of Science, Fayoum University, Fayoum, Egypt; 2 Department of Pediatrics, Faculty of Medicine, Fayoum University, Fayoum, Egypt; 3 Medical Biochemistry and Molecular Biology Department, Faculty of Medicine, Fayoum University, Al Fayoum, Fayoum, Egypt

**Keywords:** Type 1 diabetic mellitus, miR-410, miR-133, miR-582

## Abstract

**Background:**

Diabetes mellitus (DM) is a complex metabolic disease with numerous consequences that are especially linked to it. Because microRNAs (miRNAs) are produced specifically in the pancreas and remain stable in various bodily fluids, they can be used for early diagnosis as well as tracking the course and severity of the disease. This study examined the serum expression of miR-410, miR-133, and miR-582 in Egyptian patients with Type 1 Diabetes Mellitus.

**Methods:**

There were two groups of 120 participants in this study: 40 healthy children and 80 diabetic children. Samples of venous blood were obtained from every participant. The quantitative real-time PCR (qRT-PCR) method was used to determine the serum expressions of miR-410, miR-133, and miR-582.

**Results:**

Our findings showed a statistically significant difference in the median relative expression levels of miR-410, miR-133, and miR-582 between the T1DM group and the control group. T1DM patients had higher levels of miR-410 (*P* < .0001), miR- 133 (*P* = .006), and miR-582 (*P* < .0001) compared to controls. ROC curve analysis indicated that miR-410, miR-133, and miR-582 can distinguish T1DM patients from healthy subjects. For miR-410, at a cutoff point of 0.0009, sensitivity was 62%, specificity was 80%, and the area under the curve (AUC) was 0.713. For miR-133, the cutoff was 0.0016, with AUC 0.628, sensitivity 63%, and specificity 60%. For miR-582, the cutoff was 0.00028, with AUC 0.668, sensitivity 71.0%, and specificity 50%.

## Introduction

Type 1 diabetes mellitus (T1DM) is an autoimmune disease linked to insufficient insulin production and is brought on by the dysregulation of pancreatic islet b-cells, which is mediated by T-cells [1]. The World Health Organization estimates that 8.4 million people worldwide suffered from type 1 diabetes in 2021, making it one of the most prevalent chronic diseases affecting children [2].

The interplay of environmental, genetic, and epigenetic variables causes type 1 diabetes, an immune-based illness [3]. The initial sign of b-cell autoimmunity is the existence of autoantibodies [4]. Analysis of the presence of autoantibodies against islet antigens, including those against islet cells (ICA), glutamate decarboxylase (GADA), insulin (IAA), tyrosine phosphatases (IA-2 and IA-2b), and zinc transporter 8 (ZnT8), is currently the accepted approach for identifying those at risk for type 1 diabetes [5].

Certain miRNAs may be very useful in clinical settings for both diagnostic and complication prediction [6, 7]. They can be actively secreted by cells or during tissue damage and they have been employed as biomarkers for immune activity changes and beta cell renewal or death [8].

Numerous studies have shown that miRNAs have a part in the pathophysiology of type 1 diabetes, mainly by interfering with the activity, secretion, and synthesis of insulin by pancreatic β-cells. They are also known to aid in the production of autoreactive agents, which contributes to the decomposition of β-cells [9]. These miRNAs link T1D pathophysiology to the pathways of glycosaminoglycan production, axon guidance signalling, Rap1 signalling, focal adhesion, and neurotrophin signalling [10]. In order to achieve protein homeostasis, recent research has highlighted the therapeutic potential of miRNA-based approaches, such as using miRNA inhibitors to counteract overexpressed miRNAs or miRNA mimics to restore downregulated miRNAs [11]. Preclinical studies have also shown that long-term administration of particular miRNAs is safe [12].

For the studied microRNAs (miRNAs), miR-410, miR-582, and miR-133 all play unique roles in biological processes, although direct evidence linking them all specifically to the pathogenesis of Type 1 Diabetes (T1DM) is limited. Their roles mostly center on immunological control, cellular apoptosis, and stress responses; processes which are linked to the autoimmune destruction of insulin-producing beta-cells in the pancreas that is a hallmark of type 1 diabetes.

Studies have demonstrated that miR-410 plays a role in cellular stress responses and immunological modulation. *Immune control*: Low expression of miR-410 in T cells causes elevated levels of the cytokine interleukin-10 (IL-10) in the autoimmune illness systemic lupus erythematosus (SLE). This is accomplished by controlling the protein STAT3, which is involved in cell division and proliferation. Although not unique to T1DM, our study emphasizes how immune cell function, a fundamental aspect of T1DM pathogenesis, is regulated by miR-410 [13].

### Beta-cell stress

By controlling the Bcl-2/Bax pathway, elevated miR-410-5p levels in diabetic cardiomyopathy, a consequence of diabetes, encourage cell death [14]. This implies a part in triggering apoptosis in diabetics. A similar mechanism may be at play in the death of insulin-producing cells in type 1 diabetes, where inflammation and elevated glucose levels lead to severe beta-cell stress.

Advanced glycation end products (AGEs) interfere with the nitric oxide (NO) pathway in Type 1 Diabetes Mellitus (T1DM). AGEs accumulate as a result of high blood sugar and attach to endothelial cells’ receptors for AGEs (RAGE). By blocking the enzyme endothelial nitric oxide synthase (eNOS), this binding results in oxidative stress and inflammation, which lowers the amount of NO available. By affecting the NO pathway, miR-582 may act as a defense mechanism in the body against AGE-induced damage [15].

Although MiR-133 is mostly a muscle-specific miRNA, it also plays other activities that might be related to the pathophysiology of T1DM.

### Insulin resistance and glucose uptake

Patients with diabetes or insulin resistance have downregulated miR-133a in their skeletal muscle. It controls mitochondrial biogenesis, and by obstructing the mitochondrial biosynthesis pathway, its low expression may be a factor in insulin resistance in skeletal muscle. This implies that by impairing glucose metabolism in peripheral organs, a reduction in miR-133a may exacerbate the pathophysiology of type 1 diabetes [16].

### Cardiovascular complications

Diabetic cardiomyopathy may result from a substantial decrease in the cardioprotective microRNA miR-133a in diabetic hearts. This diabetes-induced cardiac fibrosis can be avoided by overexpressing miR-133a. This indicates that, while it is not the main cause of type 1 diabetes, it plays a role in treating its consequences [17].

Our aim in this study was to investigate the expression profiles of miR*-*410-3p, miR-133a-3p, and miR- 582-5p in Egyptian children with type 1 diabetes mellitus (T1DM) and to correlate between the expression levels of these specific miRNAs and the various clinical and biochemical characteristics of the patients.

## Subjects and method

There were 120 participants in this study, including 40 healthy children as controls and 80 children with type 1 diabetes. The pediatrics department’s outpatient clinics at Fayoum University Hospital in Egypt served as the source of the patients.

## Inclusion criteria for T1D patients

- Confirmed T1D Diagnosis: Participants must have a confirmed diagnosis of Type 1 Diabetes, often with evidence of autoimmune markers: Presence of one or more diabetes-related autoantibodies (e.g., anti-GAD, anti-insulin) is often an inclusion criterion to confirm autoimmune destruction of beta cells.
- Age Range: between 2 and 16 years.

## Exclusion criteria for T1D patients

### Significant organ dysfunction

Conditions like severe liver dysfunction, severe chronic kidney disease, or those requiring renal replacement therapy.

### Active infections or other comorbidities

Active infections (e.g., HIV, Hepatitis B or C), cancer, or other serious systemic conditions.

### Specific treatments

Use of certain medications, such as immunosuppressive therapy.

## Ethical considerations

The protocol for the present study was in accordance with the ethical principles of the Helsinki Declaration [18]. The study received approval with authorization number (M771) from the Fayoum University Faculty of Medicine’s Ethical Committee. The parents or legal guardians of each participant gave their informed consent to participate.

Four milliliters of venous blood were extracted from each patient and the control, and after being clotted for fifteen minutes, the samples were centrifuged for ten minutes at 4000 RPM. RNAs were extracted from sera using Qiagen (Valenica, CA, USA) extraction kits by the manufacturer’s instructions. The eluted RNA was split into two fractions: the smaller fraction (5 µL) was used for the NanoDrop spectrophotometric RNA quantification and purity assessment, while the larger component was kept at -80 °C pending further analytical procedures.The miRCURY LNA RT Kit (Qiagen, Maryland, USA) was used to reverse-transcribe the extracted RNA in a total volume of 10 ul RT reactions, following the manufacturer’s instructions.For the detection of miR-410, miR-133, and miR-582 via using quantitative real-time PCR (qPCR), a 10 µl PCR reaction mix was created using the miRCURY LNA SYBR^®^ Green Master Mix [Qiagen, Maryland, USA] and miRCURY LNA miRNA PCR Assays [Qiagen, Maryland, USA] reagents in addition to synthesized cDNA. Specific miRNA-410 primer (Cat. No. YP00204042), miRNA-133 primer (Cat. No. YP00206021), miRNA-582 primer (Cat. No. YP0020425), and the housekeeping gene miR-16-5p (Cat. No. YP00205702), which serves as a control. The qRT-PCR was set up using the cycle parameters listed below: The activation phase was conducted at 95 °C for the first 15 min. The procedure was repeated following 15 s of cycling denaturation at 94 °C, 30 s of annealing at 55 °C, and 30 s of extension at 70 °C. Forty repetitions of these operations were made. The ΔΔCt method was used to measure relative RNA expression [19].

The Statistical Package for Social Sciences (SPSS) 22 were used to analyze the data. The data was shown as median (interquartile range, or IQR), number, percentage, and mean ± standard deviation (SD). The Mann-Whitney-U test was used if the variables were not normally distributed; meanwhile the Independent-t-test was used if the variables were normally distributed in the comparison between different groups. The qualitative data was presented using numbers and percentages, and its significance was assessed using the chi-square (χ2) test. Using Pearson correlation, the correlation coefficient (r) was calculated to determine the degree of relationship between miR-410, miR-133, and miR-582 and the patients’ clinicopathological data. The cut-off points that demonstrate the markers’ maximum sensitivity and specificity were found using the ROC curve. The statistical significance was defined as P values less than 0.05.

## Results

### The demographic features of the research groups

According to the age-related demographic characteristics of the cases, there were eight cases in the early childhood phase (mean age of onset: 3.3), forty cases in the mid-childhood phase (mean age of onset: 5.43), and thirty-two cases in the early adolescence phase (mean age of onset: 8.65). Mid-childhood and early adolescence have the highest prevalence, with peaks often recorded at 5.3 years, comparable to many neighbouring nations. Less frequently, infant/toddler onset occurs [20]. Although certain regional publications from Egypt have reported a little female predominance in particular samples/periods, our data report indicates a nearly equal male: female occurrence in terms of sex ratio [20]. According to our findings, there was no statistically significant variation in the distribution of sexes or age across the study groups **(**Table 1**).**

**Table 1:** Assessment of the research groups’ demographic characteristics

		Cases					Control					*P* value
		Mean	SD	Range	*N*	%	Mean	SD	Range	*n*	%	
Age (yr)		10.4	3.7	3.0–18			9.6	3.8	4.0–16			0.32
Gender	F				39	48.8%				23	57.5%	0.32
	M				41	51.2%				17	42.5%	
BMI		18.80	4.452	12.4			19.2.	4.8	13.9–36.			0.66
Weight(Kg)		34.7	13.6	12.0			34.3	13.1	18–59.			0.86
Height(M)		134	20	86			132	21	100–172.			0.69

Abbreviations: BMI, Body mass index

According to the Egyptian paediatric T1D cohorts *not obese at diagnosis*, there were 37 patients with a normal BMI, 40 with a low BMI, and 3 with a high BMI (many are normal/low BMI z-scores or undernourished in various Upper Egypt cohorts). Nutrition issues and BMI z-score evaluations are reported in recent Egyptian case-control and clinic research [21].

In terms of consanguinity and family history, 12 out of 80 children had positive consanguinity; nevertheless, there is conflicting evidence regarding a high correlation between consanguinity and childhood T1D in Egypt. There are still many children with T1D who have no first-degree relatives that are impacted [22].

### The clinical features of the research groups

There was a highly statistically significant difference between T1DM patients and control as regards the mean values ± SD of HbA1C (*P* < .0001) and SGPT (*P* = .02) with high level in the diseased group, and platelets count (*p* = .004) with low levels in the diseased group (Table 2).

**Table 2:** Laboratory data of the research groups

	Cases		Control		*P* value*
	Mean ± SD	Median(IQR)	Mean ± SD	Median(IQR)	
HbA1C (%)	9.87 ± 2.36	10.(8–11)	11.6 ± 0.6	3.6(3.1–4.7)	< 0.001
HGB	12.1 ± 1.4	12.1(11–13)	11.2 ± 0.8	11.2(10.8–11.8)	< 0.001
WBCs	6.58 ± 3.47	5.85(4.6–7.2)	6.72 ± 2.4	5.9(4.9–8.9)	0.47
PLT	274.69 ± 96.9	271.(210–330)	234.7 ± 69.6	213.(180–262)	0.004
SGPT	19 ± 9	18 (13–20)	16 ± 7	14(11–20)	0.02

* Mann-whitney u test was done

Abbreviations: HbA1C, hemoglobin A1C: HB, Hemoglobin; WBC, White blood cell; PLT. Platelet count: SGPT, Serum Glutamic Pyruvic Transaminase

### Relative expression levels of miR- miR-410, miR-133, and miR-582 between study groups

The median relative expression levels of miR-410 (*P* < .0001), miR-133 (*P* = .006), and miR-582 (*P* < .0001) were significantly different between the T1DM and control groups, with T1DM patients exhibiting overexpression of the markers (Table 3).

**Table 3:** Evaluation of the fold change of miR-410, miR-133a and miR-582 between the research groups

	Median (IQR)	*P* value
miR-410-5p	2.48(0.91-4.47)	< 0.001
miR-133a-3p	1.72(0.65-5.78)	0.006
miR-582-5p	0.551(0.2167 − 2.205)	< 0.001

### Correlation between serum expression levels of miR-410, miR-133, and miR-582 and the clinicopathological data of the patients

Spearman correlation of expression level of miR-410, miR-133, and miR-582 showed that there was positive significant correlation between the expression level of miR-410 and age (*p* = .010), weight(*p* = .019), Height (*p* = .006), Onset of the disease (*p* = .008) Meanwhile, there was positive significant correlation between the expression level of miR-133and age (*p* = .010), Height (*p* = .007), Onset of the disease (*p* = .007) (Table 4). Also there was a positive significant correlation between the expression level of these markers in T1DM group (Table 5).

**Table 4:** Correlation between markers and age, duration and laboratory findings

		miR-410-5p	miR-133a-3p	miR-582-5p
Age (yr)	r	0.286^*^	0.285^*^	0.025
	P value	.**010**	.**010**	0.823
BMI	r	0.153	− 0.008	0.092
	P value	0.177	0.945	0.419
Weight	r	0.261^*^	0.180	0.052
	P value	.**019**	0.110	0.650
Height	r	0.302^**^	0.299^**^	0.047
	P value	.**006**	.**007**	0.682
Onset (at age:)	r	0.295^**^	0.301^**^	0.029
	P value	.**008**	.**007**	0.797
Duration	r	0.118	0.128	0.046
	P value	0.300	0.260	0.687
HbA1C (%)	r	0.004	0.064	0.069
	P value	0.972	0.579	0.547
Total Cholestrol (mg/dl)	r	− 0.096	− 0.049	0.048
	P value	0.395	0.663	0.672
HDL	r	− 0.104	− 0.168	− 0.050
	P value	0.357	0.137	0.660
LDL	r	− 0.071	− 0.009	0.016
	P value	0.530	0.940	0.886
TGs (mg/dl)	r	0.157	− 0.015	0.026
	P value	0.165	0.894	0.820
TSH (mIU/L)	r	− 0.148	− 0.155	− 0.009
	P value	0.191	0.169	0.935
Free T4 (ng/dL)	r	− 0.063	− 0.112	− 0.114
	P value	0.576	0.324	0.315
HGB% (gl/dl)	r	− 0.070	− 0.056	− 0.181
	P value	0.538	0.624	0.107
WBCs	r	− 0.098	− 0.096	− 0.094
	P value	0.389	0.398	0.407
PLT	r	0.050	0.076	0.020
	P value	0.662	0.506	0.862
SGPT (IU/L)	r	0.148	0.134	0.202
	P value	0.191	0.236	0.073
Alb/Creat Ratio	r	0.148	− 0.008	0.060
	P value	0.191	0.947	0.596

**Table 5:** Correlation between the markers

		miR-410-5p	miR-133a-3p	miR-582-5p
miR-410-5p	r	1.000	0.610^**^	0.598^**^
	P value	.	**0.000**	**0.000**
miR-133a-3p	r	0.610^**^	1.000	0.515^**^
	P value	**0.000**	.	**0.000**

**. Correlation is significant at the 0.01 level (2-tailed)

### Sensitivity and specificity of miR-410, miR-133, and miR-582 receiver operating characteristics

**Fig. 1:**
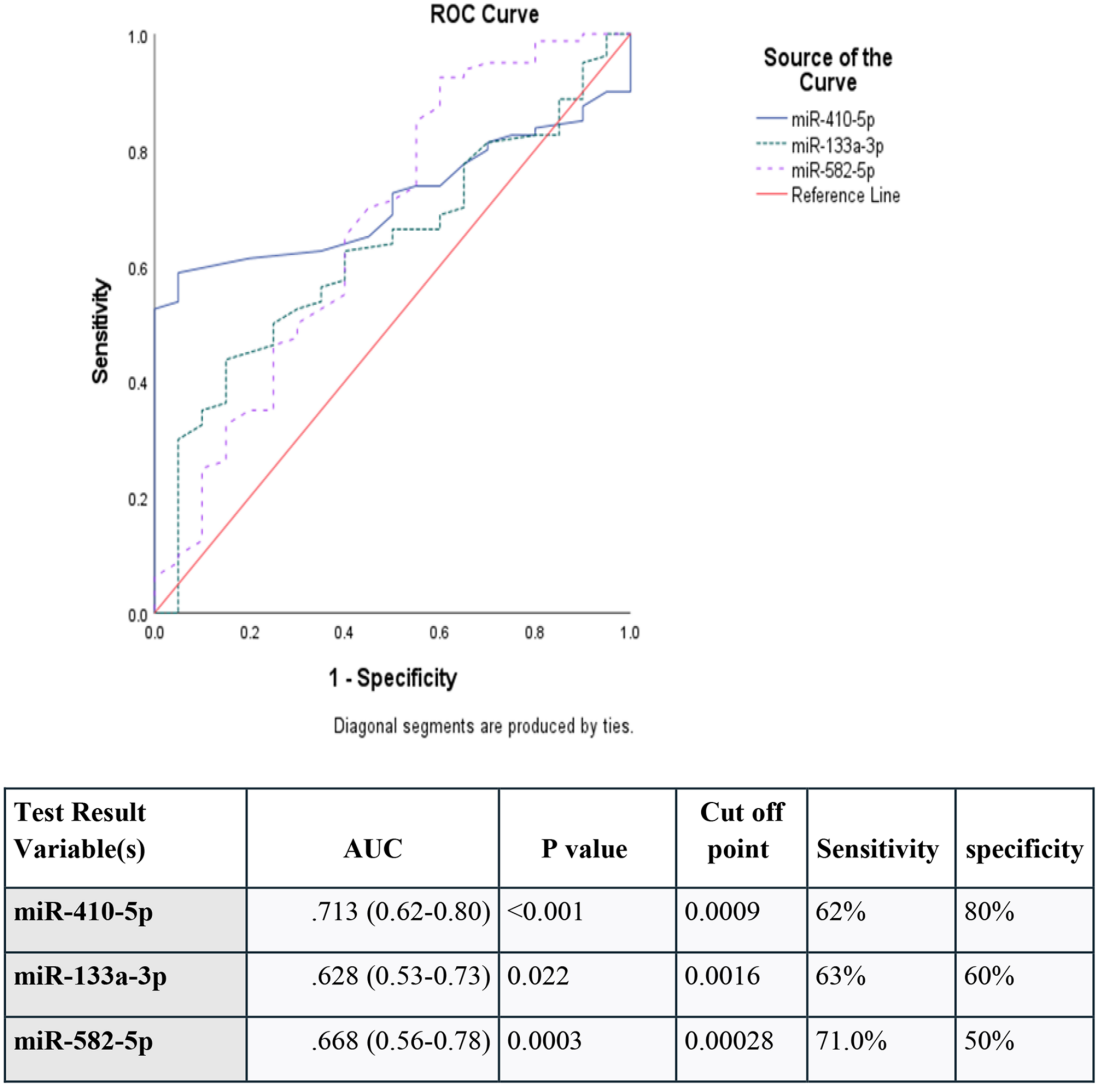
Receiver operating characteristic of sensitivity and specificity of miR-410, miR-133 and miR-582

According to the ROC curve, miR-410, miR-133, and miR-582 can distinguish between individuals with type 1 diabetes and healthy individuals. At a cutoff of point 0.0009, the sensitivity of miR-410 was 62%, the specificity was 80%, and the AUC was. 713. At a cut-off point 0.0016for miR-133 (AUC = 0.628 with a 63% sensitivity and 60% specificity) and at a cut-off of 0.00028 for miR-582 (AUC = 0.668 with a 71.0% sensitivity and 50% specificity) (Fig. 1).

### Stepwise multivariate logistic regression showing the predictors of the markers

Multivariate logistic regression analysis identified HGB (*P* = .027), WBCs (*P* = .011), SGPT (*P* = .012), and Alb/Creat Ratio (*P* < .001 as predictors for miR-410 (Table 6). Alb/Creat Ratio (*P* = .005) as a predictor for miR-133 (Table 7). HGB. (*P* = 015) and Alb/Creat Ratio (*P* = 004) as predictors for miR-582-5p (Table 8).

**Table 6:** Predictors of miR-410-5p stepwise regression

Model	Unstandardized coefficients		Standardized coefficients	t	Sig.
	B	Std. Error	Beta		
(Constant)	1.792	10.653		0.168	0.867
Age (yr)	− 0.996	2.069	− 0.414	− 0.481	0.632
BMI	0.082	0.174	0.042	0.473	0.638
Onset (at age:)	1.530	2.105	0.515	0.727	0.470
Duration	1.452	2.106	0.432	0.690	0.493
HbA1C (%)	0.379	0.356	0.103	1.065	0.291
Total Cholestrol (mg/dl)	− 0.044	0.036	− 0.170	-1.226	0.225
HDL	0.058	0.075	0.105	0.763	0.448
LDL	0.052	0.041	0.178	1.293	0.201
TGs	0.034	0.020	0.198	1.747	0.086
TSH	− 0.027	0.044	− 0.051	− 0.599	0.551
Free T4(ng/dL)	− 0.346	0.888	− 0.047	− 0.389	0.698
HGB% (gl/dl)	-1.548	0.681	− 0.233	-2.272	.**027**
WBCs	0.633	0.240	0.256	2.634	**0.011**
PLT	− 0.012	0.009	− 0.133	-1.321	0.191
SGPT(IU/L)	0.242	0.093	0.239	2.600	**0.012**
Alb/Creat Ratio	0.053	0.012	0.457	4.531	**0.000**

a. Dependent Variable: miR-410-5p

**Table 7:** Predictors of miR-133a-3p stepwise regression

Coefficients^a^					
Model	Unstandardized coefficients		Standardized coefficients	t	Sig.
	B	Std. error	Beta		
(Constant)	2.434	9.732		0.250	0.803
Age (yr)	− 0.983	1.890	− 0.585	− 0.520	0.605
BMI	− 0.281	0.159	− 0.208	-1.769	0.082
Onset (at age:)	1.545	1.923	0.745	0.804	0.425
Duration	1.330	1.924	0.566	0.691	0.492
HbA1C (%)	0.290	0.325	0.112	0.890	0.377
Total Cholestrol (mg/dl)	0.019	0.033	0.103	0.568	0.572
HDL	− 0.013	0.069	− 0.033	− 0.182	0.857
LDL	− 0.021	0.037	− 0.102	− 0.568	0.572
TGs	− 0.015	0.018	− 0.124	− 0.833	0.408
TSH	− 0.022	0.040	− 0.061	− 0.544	0.589
Free T4(ng/dL)	− 0.301	0.811	− 0.058	− 0.372	0.712
HGB	− 0.050	0.622	− 0.011	− 0.081	0.936
WBCs	− 0.012	0.220	− 0.007	− 0.055	0.956
PLT	0.001	0.008	0.020	0.154	0.878
SGPT(IU/L)	0.023	0.085	0.033	0.274	0.785
Alb/Creat Ratio	0.031	0.011	0.385	2.921	.**005**

^a^. Dependent Variable: miR-133a-3p

**Table 8:** Predictors of miR-582-5p stepwise regression

Model	Unstandardized coefficients		Standardized coefficients	t	Sig.
	B	Std. Error	Beta		
(Constant)	21.365	11.999		1.781	0.080
Age (yr)	-1.128	2.330	− 0.503	− 0.484	0.630
BMI	0.019	0.196	0.011	0.099	0.922
Onset (at age:)	0.962	2.371	0.347	0.406	0.686
Duration	1.567	2.372	0.500	0.660	0.512
HbA1C (%)	0.517	0.401	0.150	1.288	0.203
Total Cholestrol	0.051	0.040	0.213	1.271	0.209
HDL	− 0.143	0.085	− 0.279	-1.681	0.098
LDL	0.004	0.046	0.016	0.094	0.925
TGs	− 0.023	0.022	− 0.143	-1.040	0.302
TSH	− 0.059	0.050	− 0.123	-1.187	0.240
Free T4(ng/dL)	0.568	1.000	0.082	0.568	0.572
HGB	-1.931	0.767	− 0.312	-2.516	.**015**
WBCs	− 0.035	0.271	− 0.015	− 0.129	0.898
PLT	− 0.009	0.010	− 0.108	− 0.890	0.377
SGPT(IU/L)	0.189	0.105	0.200	1.803	0.076
Alb/Creat Ratio	0.040	0.013	0.370	3.040	.**004**

a. Dependent Variable: miR-582-5p

## Discussion

MicroRNAs have been identified as important modulators of beta-cell function and autoimmune responses in type 1 diabetes (T1D) research. Dysregulated miRNAs are implicated in the aetiology, progression, and complications of the disease, including retinopathy and nephropathy [23]. These tiny RNA molecules may be used as therapeutic targets for novel therapies meant to maintain beta-cell function and slow the progression of disease, as well as possible diagnostic indicators for early identification. Peripheral blood mononuclear cells (PBMC) exhibit distinct miRNA expression patterns that may serve as helpful indicators for T1D tracking and diagnosis. The evidence provided highlights the significance of dysregulated miRNAs in the genesis and etiology of type 1 diabetes. Studies by Takahashi et al. [24], Yang et al. [25], and Massaro et al. [26] show that distinct miRNA expression patterns can identify individuals with type 1 diabetes, linking these molecules to important pathways like insulin signaling, immunological control, and diabetic complications.

Our findings revealed a significant difference in the median relative expression level of miR-410 between the T1DM and control groups with upregulation of the marker in T1DM patients, Targets like vascular endothelial growth factor (VEGFA), insulin-like growth factor (IGF-1) receptor, and serine/threonine kinase 1 (AKT1) were linked to this miRNA, according to the KEGG pathway enrichment analysis [27]. VEGF is linked to the development of neuropathy and nephropathy in diabetes and is a major promoter of proliferative diabetic retinopathy [28]. The IGF-1 receptor regulates glucose and cell growth and may cause pathological conditions including cancer and cardiovascular disease [29]. AKT1 has a key role in the control of β-cell mass and many insulin metabolic processes [30]. Additionally, Zou et al. discovered that miR-410-5p targeted Smad 7 to trigger the TGFβ signaling pathway [31].

Our findings also revealed that the median relative expression level of mir-133 was significantly different between the T1DM and control groups with T1DM patients exhibiting higher levels of the marker. According to Gong et al. miR-133 can directly reduce insulin-like growth factor (IGF-1) receptor expression through translational repression [32]. Furthermore, by targeting the Krueppel-like factor 15 (Klf15) mRNA, miR-133a has been shown to indirectly affect GLUT4 expression by inhibiting this transcriptional factor, which increases GLUT4 expression. This results in a decrease in GLUT4 expression as well as insulin-stimulated glucose uptake [33].

Additionally, we demonstrated that the median relative expression level of miR-582-3p differed significantly between the T1DM and control groups with up regulation of the marker in T1DM patients, Diabetes causes decreased nitric oxide (NO) production, which contributes to endothelial dysfunction. The AGEs/RAGE interaction causes endothelial dysfunction and an elevated risk of cardiovascular illnesses by lowering NO and eNOS (endothelial Nitric Oxide Synthase) levels. Numerous miRNAs contribute to the regulation of the NO pathway [34]. MiR-185 downregulation raises ROS production and lowers NO levels in diabetic mice. These effects can be undone using Huayu Tongmai Granules [35]. Nitric oxide synthase 3 (NOS3) expression and NO levels are impacted by MiR-582 overexpression in deep vein thrombosis, which contributes to the pathophysiology [36].

Stepwise multivariate logistic regression demonstrates that Alb/Creat ratio is a common predictor for the three indicators. In people with type 1 diabetes, kidney damage, a common consequence of diabetes, is detected by a urine albumin-to-creatinine ratio (ACR) test. An ACR of less than 30 mg/g is considered normal, whereas microalbuminuria, which may be a sign of early kidney disease, is indicated by values between 30 and 300 mg/g. Increased ACR values may be a sign of more serious renal injury.

## Conclusion

Serum levels of miR-410, miR-133, and miR-582 may be useful diagnostic markers for Type 1 Diabetes Mellitus.

These altered miRNA profiles offer potential as diagnostic biomarkers for early detection and prognostic markers of T1D, and also present as therapeutic targets for future treatments aiming to restore beta-cell function and slow disease progression. As precision medicine advances, more advancement in this field of research may result in better treatment strategies.

## Cross-population comparisons

### Increasing prevalence

From 1990 to 2019, the number of individuals 65 and older with T1DM nearly tripled, rising from 1.3 million to 3.7 million, a 180% rise.

### Sociodemographic impact

This growth has been noted in all subgroups of the sociodemographic index (SDI), with the countries with the medium SDI showing the largest increase [37].

### Regional differences

Additionally, there are notable geographical differences in T1DM trends and genetic profiles, although information for particular areas is limited [38].

### Genetic predisposition

To categorise diabetes kinds and comprehend vulnerability to type 1 diabetes, a genetic approach is employed. Research compares groups with high and low genetic susceptibility [39].

### Autoimmunity

The autoimmune process that results in type 1 diabetes is characterised by the immune system attacking the pancreatic cells that produce insulin.

### Misclassification

Differentiating Type 1 Diabetes from Type 2 Diabetes (T2DM) properly is a major problem in cross-population comparisons. Because T2DM is far more prevalent, misclassification can distort prevalence numbers.

### Data scarcity

To comprehend the unique patterns and risk factors of type 1 diabetes in various communities and ancestries, more research is required [38].

## Limitation of the study

- More research involving a greater number of DM patients is required to increase the current database of potential miRNAs that could be used as biomarkers and offer strong support for their clinical application.
- A deeper comprehension of the functions and applications of miRNAs in DM may broaden the range of possible treatment approaches.
- Although qRT-PCR is an effective method for measuring gene expression, there are a number of biases and difficulties with it at every stage, from sample collection to data interpretation. If these issues are not appropriately addressed, they may produce inconsistent and untrustworthy outcomes. The reverse transcription stage, amplification efficiency, primer and probe design, normalisation techniques, and sample quality are the main sources of constraints.

## Data Availability

All relevant data are included in the article.
